# From Diagnosis to Delivery: Navigating Pregnancy With an Isthmocele

**DOI:** 10.7759/cureus.66182

**Published:** 2024-08-05

**Authors:** Sonali Hargunani, Nikita Bhattacharjee, Himadri Bal, Dipak Kolate, Varshini Vadithala

**Affiliations:** 1 Obstetrics and Gynaecology, Dr. D. Y. Patil Medical College, Hospital & Research Centre, Pune, IND

**Keywords:** management strategies, abnormal uterine bleeding, ceserean scar defect, high-risk pregnancy, isthmocele

## Abstract

Isthmocele is a myometrial defect in the uterine isthmus, often resulting from previous caesarean sections. With rising cesarean rates globally, including a significant increase in India, the prevalence of isthmocele has become a noteworthy clinical concern. Isthmocele can lead to symptoms such as abnormal uterine bleeding, dysmenorrhea, and secondary infertility, often detected through transvaginal ultrasound or MRI. Additionally, it can lead to caesarean scar pregnancy, a serious complication. The condition necessitates treatment, particularly in symptomatic cases or those planning future pregnancies. Early diagnosis and appropriate management are crucial for preventing complications and ensuring positive pregnancy outcomes. Here, we report a case that underscores the potential for successful pregnancy outcomes despite the presence of isthmocele, highlighting the need for tailored management strategies in such high-risk cases.

## Introduction

An isthmocele is a myometrial abnormality due to a localized thinning and outpouching of the anterior wall of the uterine isthmus. It is generally seen above a prior Caesarean scar. The incidence of Caesarean scar defect rises with several prior Caesarean deliveries. Current statistics suggest that around 60% of individuals develop an isthmocele after their first Caesarean section (C-section), increasing up to 100% after three C-sections [1]. According to the National Family Health Survey (NFHS-4), 17% of live births in the Indian subcontinent in the five years preceding the survey were delivered via C-section. Additionally, 45% of these C-sections were planned after the onset of labor pains. The prevalence of C-section deliveries in India was 8.5% in NFHS-3, but data from NFHS-4 indicate that this rate has increased to 17.2%, marking an increase of almost 9% over 10 years [2].

While an isthmocele may remain asymptomatic, it has the potential to lead to unfavorable symptoms such as dysmenorrhea and abnormal uterine bleeding. Typically, at the time of the menstrual phase, a minimal quantity of blood accumulates within the pouch-like defect, inciting an inflammatory response. The accumulation and subsequent reaction can be taken into consideration, in some ways, as the main basis of abnormal uterine bleeding (particularly post-menstrual spotting), and dysmenorrhea (because of the effect of inflammatory cytokines on perilesional nerve fibers). Secondary infertility resulting from disrupted embryo implantation triggered by an immune shift toward inflammation at the maternal-embryonal interface has also been reported [3]. Additionally, an isthmocele can heighten the risk of Caesarean scar pregnancy, a possibly fatal condition necessitating precise treatment to prevent severe uterine bleeding.

In this report, we present a case of pregnancy that was effectively managed despite the patient's diagnosis of an isthmocele.

## Case presentation

**Figure 1:**
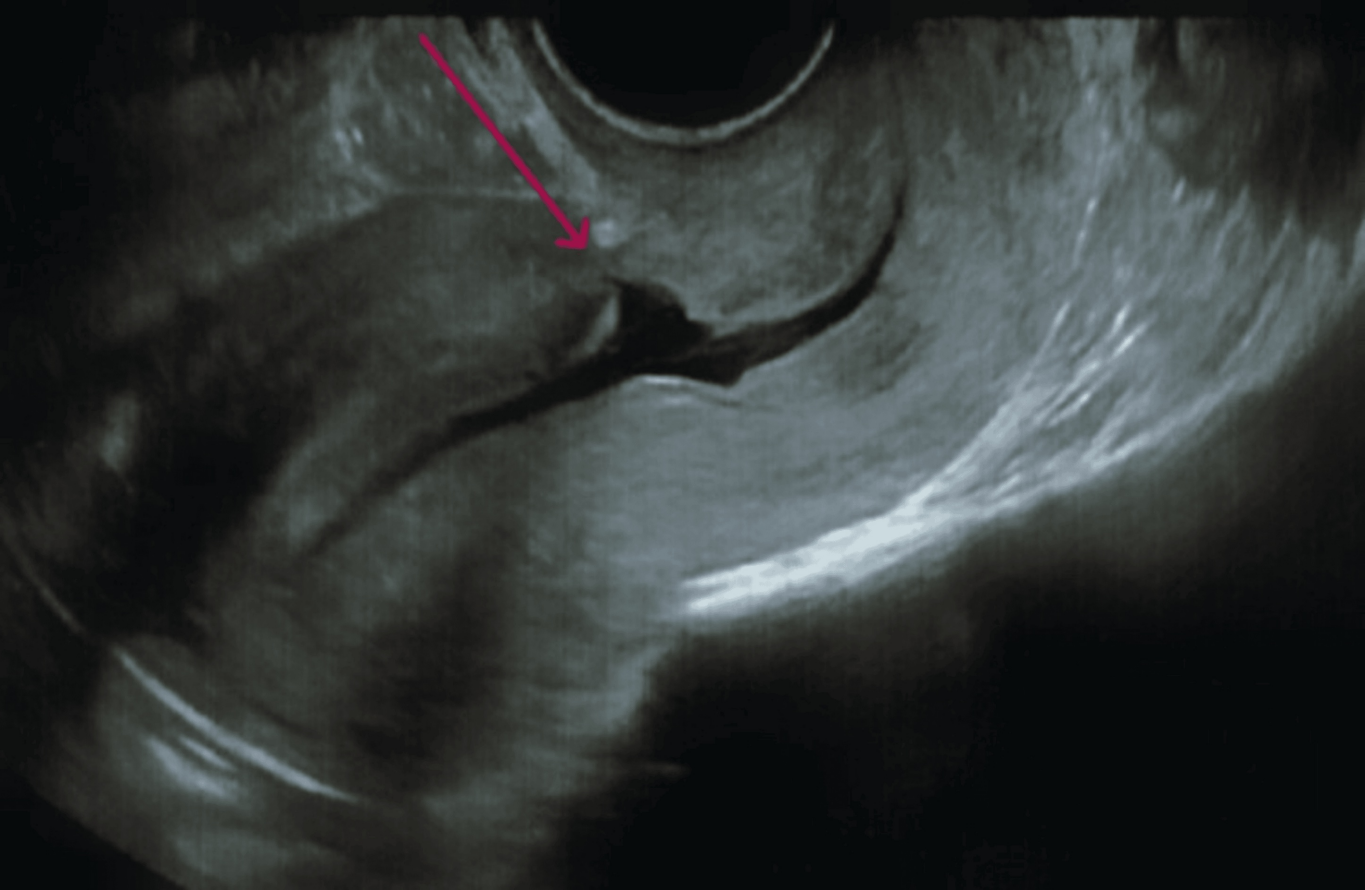
Ultrasonography showed a V-shaped defect noted at the site of the prior caesarean scar, measuring 24 x 6 x 6 mm, with a myometrial thickness of 2 mm, with the base at the endometrial cavity and apex towards the anterior wall of the isthmus, suggestive of an isthmocele.

A 28-year-old woman (para 2, living two, abortion one) presented to the outpatient department with complaints of spotting per vaginum persisting since her recent C-section delivery, which was performed two months prior. Her first child was also delivered via C-section five years ago. The patient was hemodynamically stable on examination (pulse of 86/minute, BP of 120/80 mmHg, SpO_2_ of 100% on room air, respiratory rate of 14/minute). Abdominal palpation was unremarkable. Initial ultrasound examination showed no abnormalities. Symptomatic management was done with tranexamic acid given orally as and when the patient presented with spotting per vaginum. The patient was also started on prophylactic antibiotics - T. doxycycline 100 mg twice a day (BD) and T. metronidazole 500 mg three times a day (TDS) for 14 days as a measure to prevent pelvic inflammatory disease. Despite the initial treatment, the patient continued to experience the same complaints over the subsequent months. After four months without relief, a repeat ultrasound revealed an isthmocele at the Caesarean scar site, measuring 24 x 6 x 6 mm with a myometrial thickness of 2 mm (Figure 1). The patient was counseled regarding the defect and potential complications. Surgical correction was recommended, but the patient deferred treatment.

Four months later, the patient presented with amenorrhea for two months. Repeat ultrasound showed a single live intrauterine gestation of eight weeks, distant from the isthmocele site. Despite counseling about possible complications, the patient opted to continue the pregnancy. Close monitoring was maintained throughout the pregnancy with repeated scans to ensure fetal safety and monitor for isthmocele-related complications.

**Figure 2:**
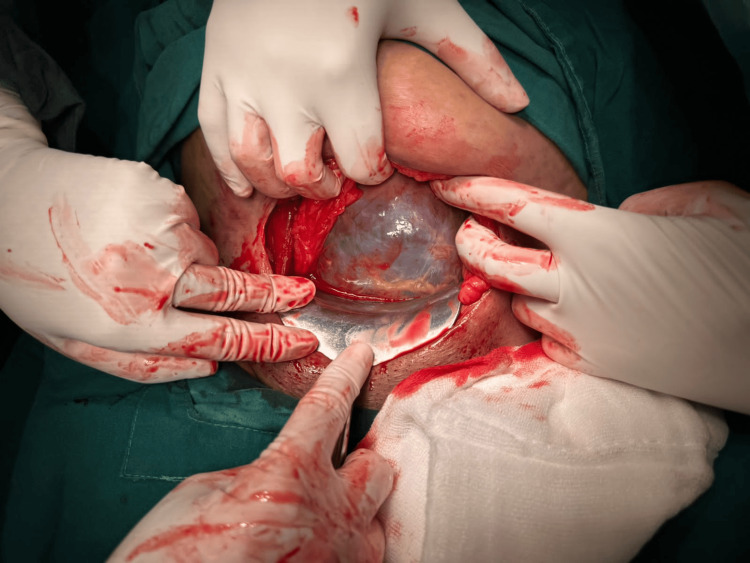
Thinned-out lower uterine segment with the baby's head visible.

**Figure 3:**
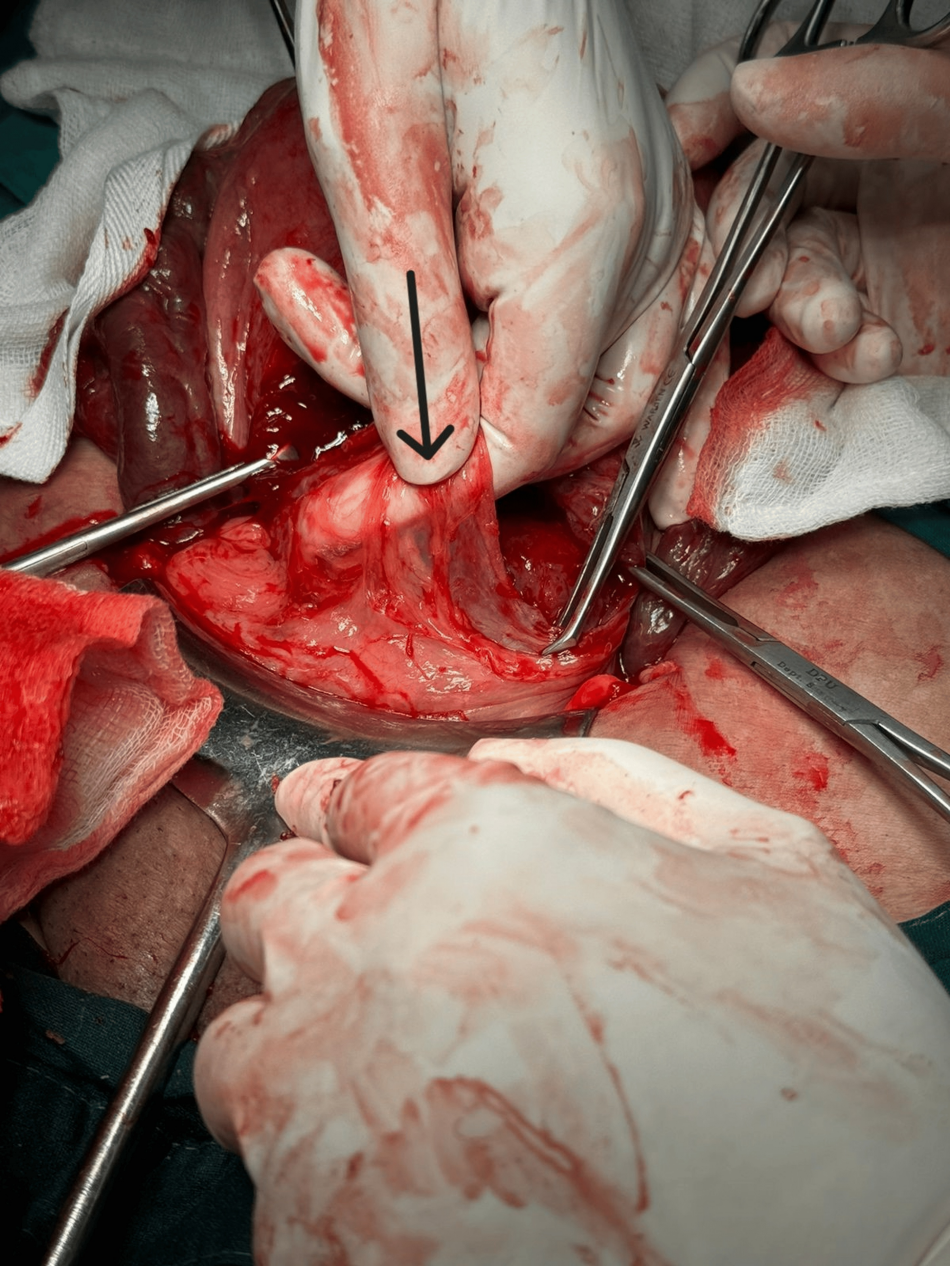
Thinned-out lower uterine segment.

At 37 weeks of gestation, the patient was posted for an elective C-section due to her history of two previous lower-segment C-sections and the presence of an isthmocele to minimize the risk of uterine rupture. As the parietal peritoneum layer was opened, the lower uterine segment was visualized. The lower uterine segment was remarkably thinned out and almost translucent with the baby's head visible, so much so that it appeared as the thin amniotic membrane, which is usually seen after the transverse incision taken on the lower uterine segment (Figure 2). During the procedure, a male infant weighing 2.5 kg was safely delivered. As the lower uterine segment was notably thinned out (Figure 3), meticulous single-layer suturing was performed on the uterus. The abdomen was closed in layers, and hemostasis was achieved. The patient received postoperative care according to protocol and was discharged on postoperative day seven.

## Discussion

In recent times, with the surge in the incidence of C-sections worldwide, there has been an increased emergence of post-Caesarean complications, both obstetric and gynecological. Isthmocele or “Caesarean scar defect” is one such complication.

Isthmocele was first explained by Hugh Morris in the year 1995, after studying cases of 51 hysterectomy specimens to describe the pathological changes of the Caesarean scar [4]. Vervoort et al. presented four distinct hypotheses regarding the formation of a uterine isthmocele. These include (1) an incision at the lower cervix when cervical dilation exceeds 5 cm, (2) deficient surgical closure, (3) the formation of adhesions between the anterior abdominal wall and the incision site, and (4) patient-dependent factors impacting the wound healing and hemostasis [5].

The detection of a uterine isthmocele is typically achieved through transvaginal ultrasound. These defects appear as anechoic triangular lesions that either communicate with the endometrial cavity or manifest as deformities in the anterior wall of the uterus. Sonohysterography can be utilized, although it can lead to an overestimation of the defect size due to increased intrauterine pressure while the procedure is ongoing. The anterior myometrium of the lower uterine segment appears weakened, with a defect covered by a thin serosal layer forming a pouch or niche. This niche typically communicates with the endometrial cavity and may appear to contain blood products. Occasionally, an isthmocele can be observed by coincidence during hysterosalpingography as contrast outpouchings at the level of the isthmus, although accurate evaluation is challenging. MRI demonstrates similar findings, particularly well-depicted on T2-weighted images. Measurements of the isthmocele's width, depth, and length are crucial for pre-surgical planning and are accurately obtained through MRI in three planes; additionally, MRI aids in ruling out other pathologies potentially related to the patient's symptoms. While a uterine isthmocele is readily identifiable via ultrasound, MRI may be employed when necessary [6].

Treatment of an isthmocele is necessary for symptomatic patients and may also be considered for asymptomatic individuals planning future pregnancies. Minimally invasive procedures are preferred for closing the isthmocele, which can be performed via hysteroscopy and laparoscopy. Research indicates that conservative management is generally ineffective, and the primary approach to managing isthmocele is minimally invasive resection, yielding optimal therapeutic outcomes [7]. Smaller defects are typically addressed through hysteroscopic repair, which are minimally invasive and less troublesome procedure. However, large defects with thin myometrium require laparoscopic repair, involving resection of the isthmocele and closure of the pouch in multiple layers. Combined hysteroscopic and laparoscopic repair offers the benefits of both approaches, reducing complications and achieving optimal repair outcomes [8,9].

It can also be hypothesized that, in late pregnancy, an undiagnosed isthmocele can be a reason for scar dehiscence. The incidence of scar dehiscence ranges between 0.2% and 4.3% of all pregnancies with a previous C-section [10].

In this unique case, opting for surgical repair of the defect was avoided due to concerns about its potential impact on the pregnancy outcome. Close monitoring with ultrasonography was done to assess the isthmocele status. Ultimately, a successful pregnancy was achieved despite the presence of the Caesarean scar defect.

## Conclusions

An isthmocele represents a significant challenge in the landscape of post-Caesarean complications, particularly as rates of Caesarean deliveries continue to rise globally. This condition, characterized by a defect in the uterine isthmus, can lead to a range of symptoms, including abnormal uterine bleeding, dysmenorrhea, and secondary infertility. Furthermore, it may elevate the risk of complications such as Caesarean scar pregnancy and uterine rupture, which necessitate vigilant management and monitoring. The case presented underscores the feasibility of achieving a successful pregnancy despite the presence of an isthmocele, especially when careful management and close monitoring are employed. The patient's decision to defer surgical correction and opt for close observation highlights the potential for positive outcomes even in high-risk scenarios. This case emphasizes the importance of individualized patient care, particularly in balancing the risks and benefits of surgical intervention versus conservative management. Future research and clinical practice should focus on refining diagnostic techniques and treatment protocols for an isthmocele to optimize patient outcomes. As the prevalence of Caesarean deliveries continues to rise, ongoing evaluation of management strategies for an isthmocele will be crucial in mitigating its impact on reproductive health and pregnancy outcomes.
